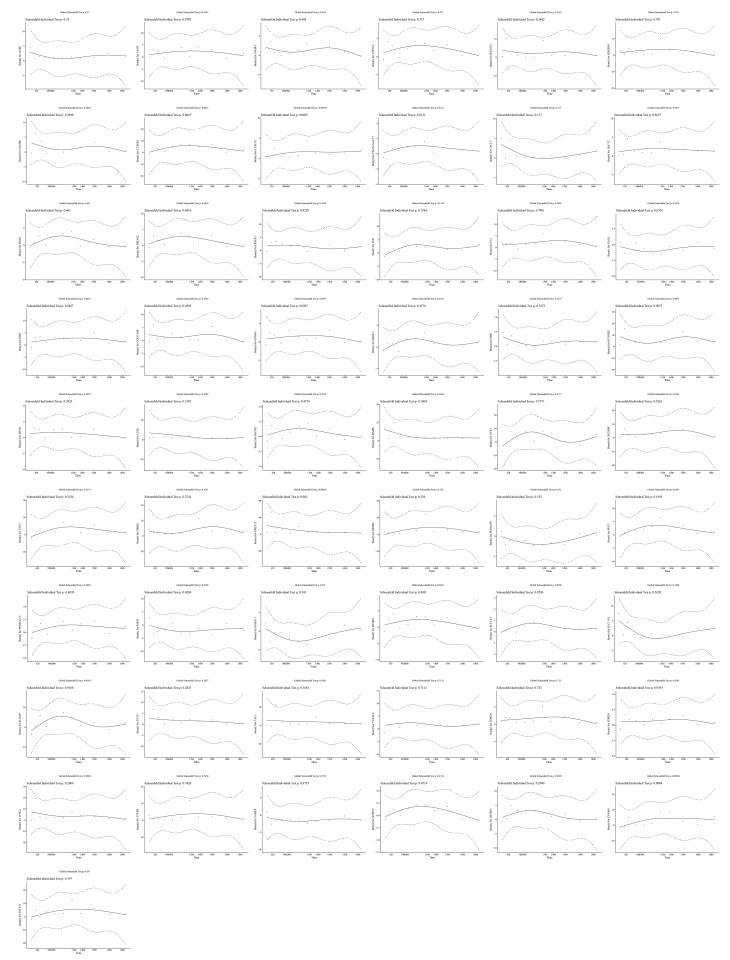# Investigating the prognostic potential of *PTPN11* gene in papillary thyroid carcinoma: A comprehensive study of bulk and single cell transcriptome

**DOI:** 10.1097/MD.0000000000046315

**Published:** 2026-05-12

**Authors:** Huiling Wang, Mian Lv, Yonghong Huang, Xiaoming Pan, Changqiang Xu, Chunyu Chen, Wuyu Tan, Huaye Lao, Minghui Qin, Hui Zhang, Guixuan Nong, Yinling Wang

**Affiliations:** a Department of Breast and Thyroid Surgery, The Second Nanning People’s Hospital, Nanning, Guangxi, PR China; b Department of Breast and Thyroid Surgery, The Third Affiliated Hospital of Guangxi Medical University, Nanning, Guangxi, PR China.

**Keywords:** pan-cancer, papillary thyroid carcinoma, prognostic risk model, *PTPN11*, single-cell transcriptome

## Abstract

PTPN11 is closely associated with cancer progression. This study aimed to explore its prognostic potential in papillary thyroid carcinoma (PTC) and identify additional PTPN11-related prognostic genes, thereby providing novel insights for PTC treatment. Bulk and single-cell transcriptomic data from public databases were utilized to perform pan-cancer analysis for mining the oncogenic potential of PTPN11, accompanied by functional enrichment, immune infiltration, and drug molecular docking analyses. Univariate Cox regression and least absolute shrinkage and selection operator analyses were employed to screen prognostic genes and construct a risk model. Finally, single-cell level analysis was conducted to identify PTPN11-related key cells and their communication patterns. Pan-cancer analysis revealed that PTPN11 expression levels (high vs low) were significantly correlated with survival differences in PTC and other cancers (*P* < .05). PTPN11 was enriched in the ribosome and oxidative phosphorylation pathways and negatively correlated with CD56 bright natural killer cells (cor = −0.35, *P* < .05). It exhibited strong binding affinities with VX-11e, irinotecan, and dactinomycin. Eight prognostic genes (*ATP2C2, OPRK1, CLSTN2, AGRP, MMP8, B3GNT4, KCNMB2*, and *DACT2*) were screened out, and a robust risk model was established. Endothelial cells were identified as key cells; the occurrence of PTC reduced their quantity and affected the frequency/intensity of their interactions with mast cells. In conclusion, PTPN11 holds promise as a prognostic marker for PTC and is of great value for clinical management.

## 1. Introduction

Papillary thyroid carcinoma (PTC) represents the predominant form of thyroid cancer (THCA). The occurrence of PTC is closely related to genetic and environmental factors.^[1]^ *BRAF*^V600E^ mutations,^[2]^ *RAS* gene mutations, and *RET*/PTC rearrangements^[3]^ are frequently in PTC. Currently, the main treatment for PTC remains surgery.^[4]^ Although the overall prognosis is good, 20% to 30% of PTC cases still exhibit high-risk features such as aggressive growth, lymph node metastasis, and even distant metastasis, leading to significantly increased treatment resistance and recurrence rates.^[5]^ Given the high incidence of PTC, specific genetic changes, and the negative impact of surgical trauma on patients’ physical and psychological well-being, finding new prognostic features and key prognostic genes for PTC is still of great significance for exploring the molecular mechanisms of PTC occurrence and progression, which will facilitate personalized patient treatment.

The *PTPN11* gene is situated on human chromosome 12q24.1, encoding the SHP2 protein (Src Homology 2 Domain-Containing Phosphatase 2), which possesses multiple functional domains, including SH2 and PTPase domains.^[6]^ Through its regulation of *RAS*/*MAPK*, *PI3K*/*AKT*, and other signaling cascades, SHP2 exhibits a dual function in controlling cell proliferation/differentiation and remodeling the tumor microenvironment.^[7]^ Numerous studies report abnormal *PTPN11* expression or mutations in diverse cancers. Breast cancer exemplifies this, where elevated SHP2 levels are significantly associated with increased tumor aggressiveness, metastasis, and worse clinical outcomes (PMID: 34591414). SHP2 facilitates the proliferation, migration, and invasion of breast cancer cells through the activation of the *RAS*/ERK signaling pathway, and it is associated with the maintenance and self-renewal capabilities of tumor stem cells.^[7]^ The mechanism of *PTPN11* in PTC is not yet fully understood, and no studies have constructed *PTPN11*-related prognostic models in PTC.

Conventional transcriptomics can reveal transcriptional expression profiles at the tissue level, but it obscures the differences between individual cells.^[8]^ Single-cell transcriptomics analyzes at the single-cell level, revealing the distribution and functional status of different cell types within tissues, greatly enhancing the resolution and accuracy of the data, helping researchers uncover cellular heterogeneity and observe dynamic changes in cell states during disease progression.^[9]^ The combined analysis of both allows researchers to observe gene functions and regulatory networks from different perspectives, revealing complex molecular interactions and signaling pathways,^[10]^ and obtaining comprehensive phenotypes-to-mechanism insights to address biological questions, providing new perspectives for basic biology and disease mechanism research.

This study is based on public datasets of PTC to conduct prognostic-related research on *PTPN11* in PTC and various cancers, perform immune infiltration and drug sensitivity analyses, and obtain prognostic genes through univariate and least absolute shrinkage and selection operator (LASSO) algorithms, constructing and validating prognostic risk models, functional enrichment analyses, etc. Single-cell transcriptomic analysis is further integrated, with the aim of furnishing a novel theoretical basis and innovative methodologies for the prognosis and treatment of PTC in clinical settings.

## 2. Material and methods

### 2.1. Compliance with reporting guidelines

This study was conducted in accordance with the Transparency in Artificial Intelligence for Nature reporting guidelines. A completed Transparency in Artificial Intelligence for Nature checklist has been provided to declare the absence of AI tool usage in manuscript drafting, figure/graphic creation, or data collection/analysis. The authors assume full responsibility for all content in this work. Additionally, we followed the STARD guidelines for diagnostic accuracy study.

### 2.2. Data collection

The bulk transcriptome data of PTC patients were derived from the cancer genome atlas (TCGA) database. This study employed the TCGA-THCA dataset, which includes 496 PTC and 59 control tissue samples and their corresponding clinical data. The visit time was April 27, 2025. In addition, the single-cell dataset GSE191288 (GPL24676), which incorporated tissue samples from 6 PTC patients and 1 control, was derived from the gene expression omnibus database.

### 2.3. Pan-cancer analysis of PTPN11 gene

We assessed *PTPN11* expression across 34 cancers using TCGA, GTEx, and TARGET data. Wilcoxon tests (SangerBox; *P* < .05) compared tumor and control samples. Tumors were divided into high expression groups(HEG) and low expression groups (LEG) using the median expression value. Kaplan–Meier(K–M) survival analysis utilized the “survival” (v 3.5.3^[11]^) and “survminer” R packages (v 0.4.9^[12]^; *P* < .05, log-rank test).

Copy number variation (CNV) data, methylation data, and corresponding gene expression data of the *PTPN11* gene in different cancers were derived from the GSCA. The proportion of CNV across diverse cancer types and the correlations between *PTPN11* expression and CNV, as well as between *PTPN11* expression and methylation, were analyzed respectively (|cor| > 0.3, FDR ≤ 0.05).

### 2.4. Expression and survival analysis of PTPN11 gene

To evaluate the discriminative capacity of the *PTPN11* gene for differentiating tumor versus control samples, a Wilcoxon test was performed to investigate the disparities in *PTPN11* gene expression levels between the PTC and control within the TCGA-THCA dataset (*P* < .05). Subsequently, PTC samples were stratified into HEG and LEG on the basis of the optimal cutoff value of *PTPN11* gene expression determined in the PTC cohort. The “survival” package (v 3.5.3^[11]^) was utilized to plot the K–M curve (*P* < .05, log-rank test).

### 2.5. Gene set enrichment analysis (GSEA)

To explore the functional pathways related to *PTPN11* in *PTC*, gene set enrichment analysis (GSEA) of *PTPN11* was performed. The “c2.cp.kegg_legacy.v2024.1.Hs.symbols” gene set served as our reference. This collection was sourced from the molecular signatures database database. Within the samples of PTC in the training set, Spearman correlation analyses were performed between *PTPN11* and all the remaining genes separately using “psych” package (v 2.1.6^[13]^) to obtain the correlation coefficients. Subsequently, genes were arranged in descending order on the basis of these coefficients. The sorted data were then utilized to conduct GSEA (|normalized enrichment score > 1, FDR < 0.25, and *P* < .05) using the implementation of the “clusterProfiler” package (v 4.7.1.3^[14]^).

### 2.6. Immune infiltration analysis

The single sample gene set enrichment analysis (ssGSEA) algorithm was applied in HEG and LEG to assess the ssGSEA scores of 28 immune cells,^[15]^ with the aim of understanding the immune infiltration of different samples in the TCGA-THCA dataset. Differences in the infiltration of all 28 immune cells were then assessed using the Wilcoxon rank-sum test. This comparison specifically targeted the HEG and LEG cohorts (*P* < .05). Furthermore, Spearman correlation was carried out to investigate the connections among the differential immune cells and *PTPN11* (|cor| > 0.3 and *P* < .05) using “psych” package (v 2.1.6^[13]^).

### 2.7. Drug susceptibility prediction and molecular docking

In the TCGA-THCA dataset, PTC chemotherapeutic agents were retrieved from the genomics of drug sensitivity in cancer. Moreover, the half maximal inhibitory concentration (IC_50_) for the HEG and LEG was estimated using “oncoPredict” package (v 1.2^[16]^) to evaluate the drug susceptibility. The sensitivity of the HEG and LEG was contrasted by means of Wilcoxon test, with *P* < .05.

The top 3 drugs exhibiting the most remarkable differences in IC_50_ values between HEG and LEG in drug sensitivity analysis were selected as key drugs for molecular docking, aiming to explore *PTPN11*’s potential as a novel drug design target. The 3D structure of key drugs was first extracted from the PubChem Project database. Subsequently, the 3D structure of the protein corresponding to *PTPN11* was extracted from the universal protein database. Thereafter, the docking simulation results were visualized using Cad Lab. The binding energy was <−5 kcal/mol, indicating good binding ability.

### 2.8. Identification and functional characterization of differentially expressed genes (DEGs)

To identify DEGs1 between PTC and control samples in TCGA-THCA, “DESeq2” (v1.42.0, PMID: 25516281) was used for differential analysis, with differentially expressed gene (DEGs 1) screened by *P* < .05 and |log₂ FC| > 0.5. Moreover, DEGs 2 were screened between *PTPN11* HEG and LEG similarly. Subsequently, the “ggplot2” package (v 3.4.1^[17]^) was utilized to construct a volcano plot for visualizing the DEGs, and the “ComplexHeatmap” package (v 2.14.0^[18]^) was employed to generate a heatmap of DEGs. In addition, the “clusterProfiler” package (version 4.7.1.3, with reference to^[14]^) was employed to carry out gene ontology (GO) and Kyoto Encyclopedia of Genes and Genomes (KEGG) enrichment analyses – with a significance threshold of *P* < .05 – on the acquired DEG 1 and DEG 2, respectively. Furthermore, Spearman correlation was carried out to investigate the connections between *PTPN11* and DEGs 1 (|cor| > 0.3 and *P* < .05) using “psych” package (v 2.1.6^[13]^).

### 2.9. Identification and protein–protein interaction (PPI) of candidate genes

To further identify the candidate genes linked to *PTPN11* in PTC, the “ggvenn” package (v 0.1.9^[13]^) was used to intersect DEGs 1 and DEGs 2; the intersection genes were used as candidate genes. In addition, with the aim of investigating gene interactions at the protein level, the candidate genes were input into the STRING with a confidence score = 0.4, and the PPI network was visualized using the “Cytoscape” software (v 3.10.2^[19]^).

### 2.10. Determination of prognostic genes, development and validation of a prognostic risk model

The TCGA-THCA tumor samples were partitioned using a 7:3 ratio for prognostic risk modeling. The larger subset (70%), comprising 347 samples, served as the training set. Validation set 1 of the study consisted of three-tenths of the split tumor samples, totaling 149 specimens, while validation set 2 was formed by all 496 tumor samples from the TCGA-THCA dataset. The “survival” package (v 3.5.3^[11]^) was employed to perform univariate Cox regression analysis on the candidate genes and *PTPN11* in the training set, where genes were filtered by hazard ratio ≠ 1 and *P* < .01. To identify prognostic genes, we applied LASSO regression via 10-fold cross-validation (using the “glmnet” package, v4.1-4^[20]^) to datasets where the proportional hazards assumption was not violated (*P* > .05). Based on the identified prognostic genes, the risk model was formulated as (coef denotes the regression coefficient for each gene, and *X* its expression level):


$$\begin{array}{l} Risk\;\;\;score=\underset{i\;\;\;=1}{\overset{n}{\sum }}\;coef\;\;\;\left(gene_{i}\right)\times X\;\;\;\left(gene_{i}\right) \end{array}$$

Based on the training set median cutoff, we classified PTC patients as high-risk group (HRG) or low-risk group (LRG). The “survival” package (v 3.5.3^[11]^) was utilized to plot the K–M curve (*P* < .05, log-rank test). Furthermore, the risk score distribution maps and survival state distribution maps were plotted. “TimeROC” package was implemented to plot the receiver operating characteristic curve for the prognostic risk model. (v 0.4^[21]^). Finally, the reliability of the model was also validated on the validation set 1 and validation set 2.

### 2.11. Single cell data quality control and high-variable gene screening

To guarantee the veracity and dependability of the subsequent analysis of the data, in the single-cell dataset GSE191288, a comprehensive quality control assessment was performed on the sample data via the “Seurat” package (v 5.0.1^[22]^). The quality control criteria were established as follows: genes that were covered by fewer than 3 cells were removed through the filtering process, cells with cellular unique molecular identifier counts (nCount_RNA) surpassing 30,000 were excluded, cells with gene expression counts (nFeature_RNA) <200 or exceeding 6000 were removed, and cells with a percentage of mitochondrial genes (percent_mt) higher than 20% were also removed. To obtain genes with relatively large intercellular coefficient of variation, after data standardization, the VST method of the FindVariableFeatures function was used to extract genes with relatively large intercellular coefficient of variation. Moreover, the first 2000 highly variable genes (HVGs) with obvious fluctuations were displayed, and the results were visualized to show the top 10 genes with the largest variation.

### 2.12. Cell dimension-reduction clustering and annotation

The “ExPosition” package (v 0.4.9^[23]^) was used to perform principal component dimensionality reduction analysis on HVGs, and the outcome of data dimensionality reduction was visualized using the JackStraw function to ascertain the statistically significant principal components (*P* < .05).

Unsupervised clustering analysis was carried out using the FindClusters and FindNeighbors functions within the “Seurat” package (v 5.0.1^[22]^). Specifically, the resolution was set to 0.4 to ascertain the quantity of cell clusters. Subsequently, the cell clusters were visualized via the RunUMAP function.

To annotate the cells in the clustering analysis, Low et al^[24]^ were consulted, and the CellMarker database was additionally used to annotate the cell clusters. Subsequently, a Dotplot was generated.

### 2.13. Identification of key cells and cell communication

To identify key cells, the cell ratio in PTC and control samples in the GSE191288 dataset was first shown; cell types exhibiting marked discrepancies between the PTC and control samples were identified as differential cells (*P* < .05). Subsequently, in accordance with the expression levels of the *PTPN11* gene in the cells, the cells in which *PTPN11* gene was highly expressed were chosen as the key cells.

To explore the interactions among cells, an analysis was conducted on ligand–receptor pairs as well as molecular interactions using “CellChat” package (v 1.6.1^[25]^) within GSE191288, thereby presenting the intercell communication networks and the receptor–ligand interaction probability point plots.

### 2.14. Statistical analysis

Bioinformatics analyses were conducted by employing the R programming language (v 4.2.3). Wilcoxon testing was applied to compare both groups, considering *P* < .05 statistically significant.

## 3. Results

### 3.1. Comprehensive analysis of PTPN11 gene expression, survival association, CNV, and methylation was conducted in pan-cancer

**Figure 1.**
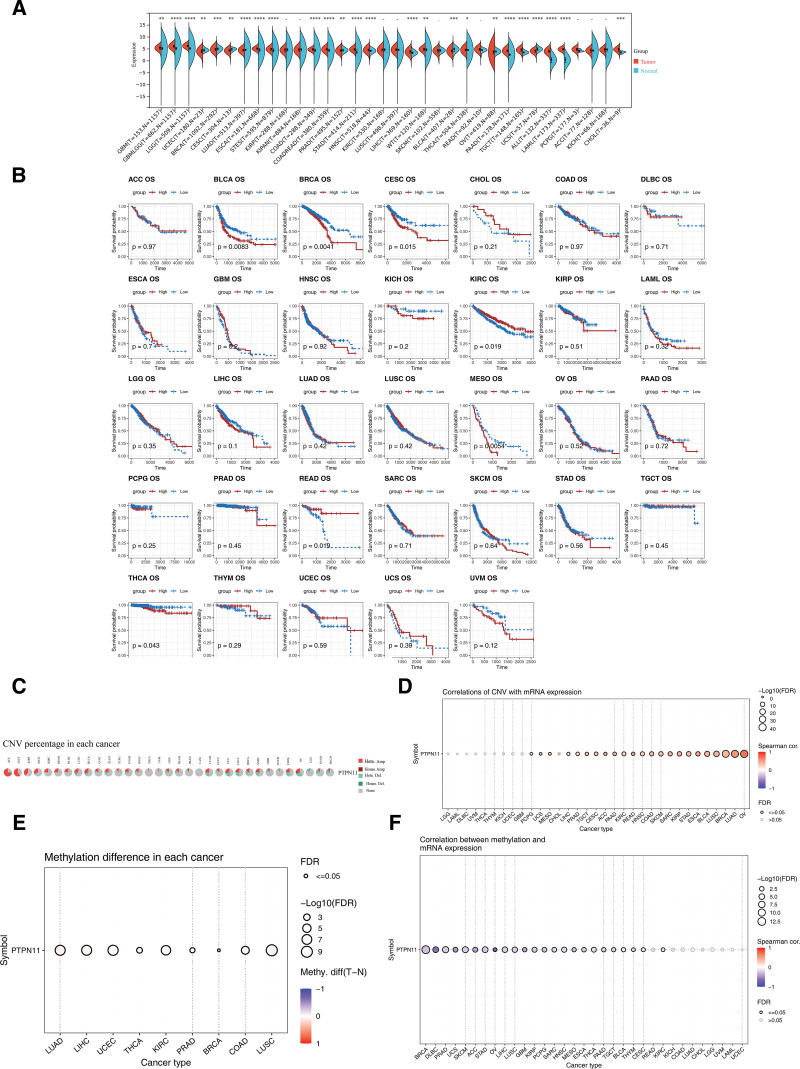
Comprehensive analysis of *PTPN11* gene expression, survival association, CNV, and methylation was conducted in pan-cancer. (A) Expression differences of PTPN11 in cancer tissues and control samples. ns: *P* > .05; *P < .05; **P < .01; ***P < .001; and ****P < .0001. (B) Survival analysis of PTPN11. Red: high expression group; blue: low expression group. (C) The proportion of CNV in different cancers. Different colors represented different types of mutations. (D) Correlation between CNV and PTPN11 expression levels. Red: positive correlation; blue: negative correlation. Hollow circles represented FDR > 0.05, while solid circles represented FDR ≤ 0.05. (E) Methylation differences between tumor samples and normal samples of *PTPN11* gene. Hollow circles represented FDR > 0.05. (F) Differences in methylation levels of *PTPN11* gene among different types of cancer. Red: positive correlation; blue: negative correlation. Hollow circles represented FDR > 0.05, while solid circles represented FDR ≤ 0.05. CNV = copy number variation.

Based on data from the SangerBox website, it was found that the expression of *PTPN11* exhibited notable discrepancies (*P* < .05) between the remaining 25 types of cancer and control samples, except for kidney renal papillary cell carcinoma, kidney renal clear cell carcinoma, rectum adenocarcinoma, kidney chromophobe, pan-kidney cohort, lung squamous cell carcinoma, skin cutaneous melanoma, pheochromocytoma and paraganglioma, and adrenocortical carcinoma (Fig. 1A). Subsequently, significant survival differences were observed in patients with bladder urothelial carcinoma (*P* = .0083), breast invasive carcinoma (*P* = .0041), cervical squamous cell carcinoma and endocervical adenocarcinoma (*P* = .015), kidney renal clear cell carcinoma (*P* = .019), mesothelioma (*P* = .0054), rectum adenocarcinoma (*P* = .019), and THCA (*P* = .043) between the HEG and LEG of *PTPN11* (Fig. 1B). These findings preliminarily indicate that PTPN11 is associated with the survival of patients with various types of cancer. Additionally, further analysis of CNV status of the *PTPN11* gene in different cancers via GSCA revealed that CNV accounted for a relatively small proportion in THCA and had no significant correlation with *PTPN11* gene expression (FDR > 0.05; Fig. 1C, D). However, the methylation level of the *PTPN11* gene exhibited notable discrepancies between THCA cancer and control samples (FDR ≤ 0.05), and methylation was significantly negatively correlated with *PTPN11* gene expression (cor < −0.3, FDR ≤ 0.05; Fig. 1E, F).

### 3.2. PTPN11 gene was involved in the regulation of PTC through diverse pathways and linked to immune cells

**Figure 2.**
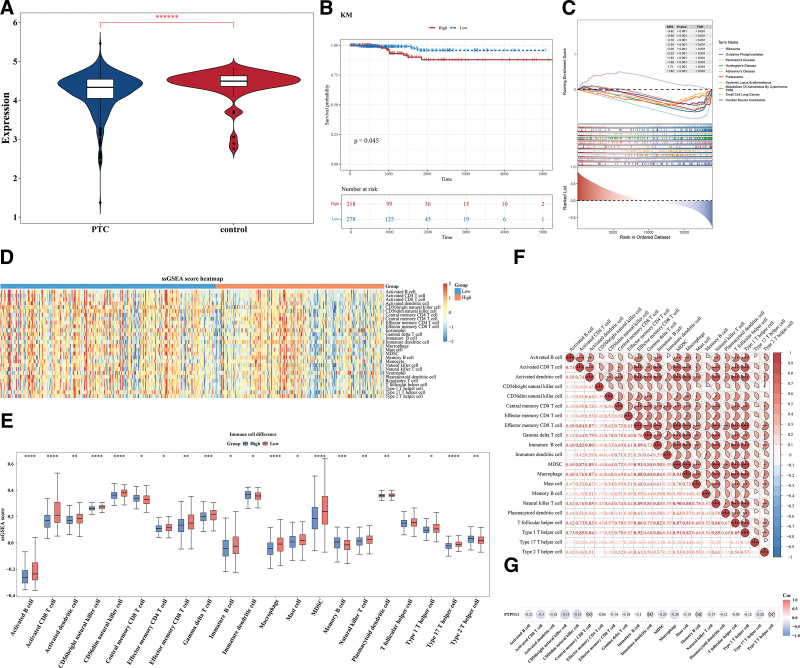
Analysis of multiple pathways involved in the regulation of PTC by *PTPN11* gene. (A) Box plot of expression for PTPN11 in disease and control groups. Blue: PTC group; red: control group. *****P < .00001. (B) Kaplan–Meier (K–M) survival curve of PTPN11 high and low expression groups. Red: high expression group; blue: low expression group. (C) GSEA enrichment analysis of PTPN11. There was a peak in the line chart, which was the enrichment score of this gene set. The genes before the peak were the core genes under this gene set. (D) Single sample gene set enrichment analysis (ssGSEA) score heatmap of immune cells. Orange: high expression group; blue: low expression group. (E) Inter group differential immune cells. Red: high expression group; blue: low expression group. *P < .05; **P < .01; ***P < .001; and ****P < .0001. (F) Correlation analysis between differential immune cells. Red: positive correlation. Blue: negative correlation. *P < .05; ***P < .001. (G) Correlation analysis between differential immune cells and PTPN11. Color represented correlation. Red: high expression; blue: low expression. GSEA = gene set enrichment analysis, K–M = Kaplan–Meier, PTC = papillary thyroid carcinoma, ssGSEA = single sample gene set enrichment analysis.

The expression of the *PTPN11* gene exhibited extremely notable discrepancies between PTC and control samples in TCGA-THCA dataset (*P* < .000001; Fig. 2A). Moreover, there were significant survival discrepancies between the HEG and LEG (*P* = .045; Fig. 2B). A total of 66 differential pathways were enriched by *PTPN11* in the TCGA-THCA tumor samples, with ribosome, oxidative phosphorylation (OXPHOS), proteasome and other pathways being significantly enriched (Fig. 2C, Table S1, Supplemental Digital Content, https://links.lww.com/MD/Q823). Overall, these outcomes furnished a robust foundation for the ongoing investigation of the molecular mechanisms of PTC.

The ssGSEA score heatmap of 28 immune cells was shown in Figure 2D. Results indicated that 21 immune cells had substantial disparities between the HEG and LEG. Compared to the control group, the HEG exhibited a significantly elevated proportion of type 2 T helper cells and a markedly reduced percentage of macrophages (both *P* < .01). These contrasting immune cell profiles suggest potential immunomodulatory effects (Fig. 2E). Among them, most immune cells showed positive correlation; Effector memory CD8 T cells and MDSC, as well as Macrophages and MDSC demonstrated the strongest positive correlation (cor = 0.93, *P* < .001; Fig. 2F). Additionally, a significant inverse correlation was observed between *PTPN11* expression and *CD56* bright natural killer (NK) cells (Spearman’s correlation coefficient = −0.35, *P* < .05), suggesting potential inhibitory effects of *PTPN11* on NK cell activity (Fig. 2G).

### 3.3. Multiple drugs might be related to PTPN11 gene in PTC

**Figure 3.**
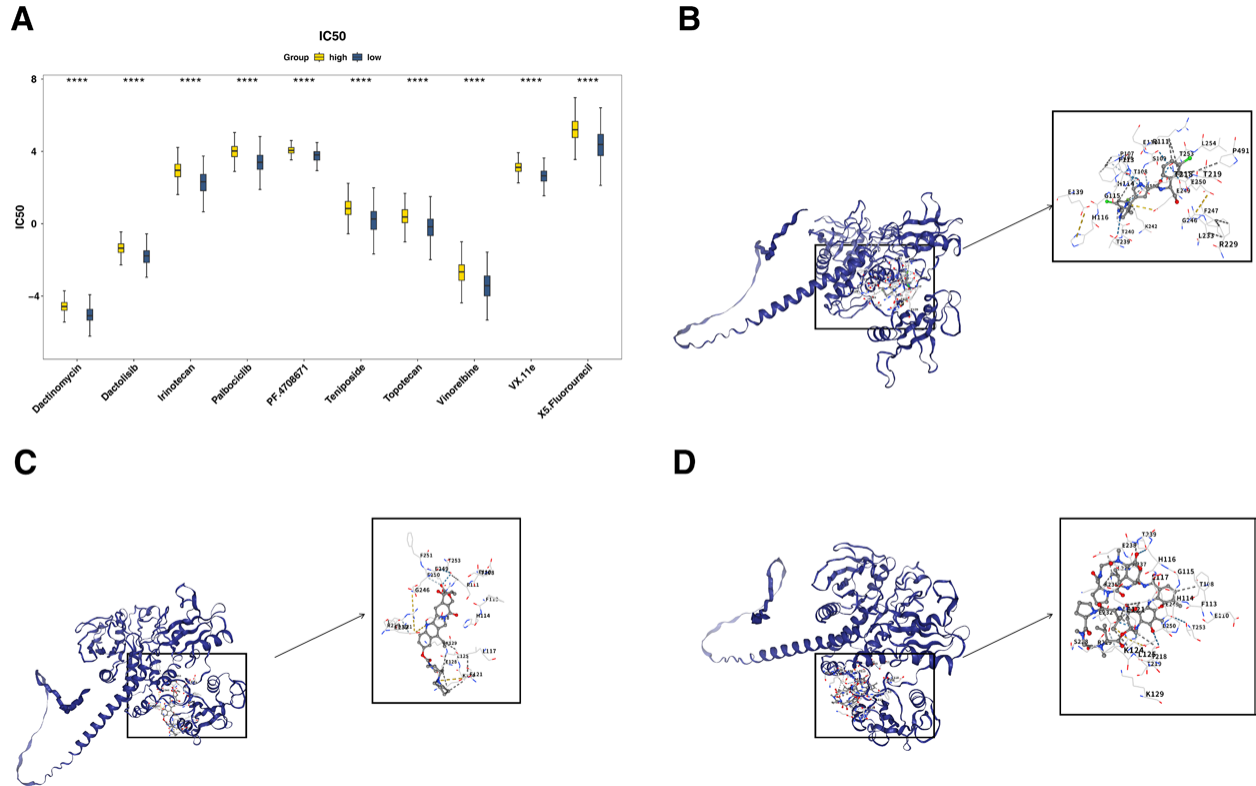
Drug prediction of *PTPN* gene. (A) Drug sensitivity analysis between high and low expression groups. Yellow: high group; blue: low group. ****P < .0001. (B–D) Molecular docking results of VX-11e, IrinotPTCan, and dactinomycin. The outermost part was the protein skeleton, with numbers represented the target of binding, blue dashed lines represented hydrogen bonds, and yellow dashed lines represented hydrophobic interactions.

The lower IC_50_ values indicated that the drug could achieve a significant therapeutic effect at a lower dose and reduce toxic and side effects. A total of 146 different drugs were detected. Among the top 10 drugs with significant differences, dactinomycin, VX-11e, irinotecan were more effective in the LEG (*P* < .0001; Fig. 3A). The binding energies in *PTPN11* and VX-11e, irinotecan, dactinomycin were −9.6, −9.7, −9.6 kcal/mol, which were <−5 kcal/mol, signifying that key genes possessed strong affinity with compounds (Table 1). The analysis of binding conformation revealed that compound active ingredients infiltrated the binding site of key genes, and there were abundant hydrogen bond donors and acceptors in the vicinity of the binding sites (Fig. 3B–D). This could potentially offer a valuable reference for the clinical management of PTC.

**Table 1:** Summary table of drug binding energy.

MOL ID	Molecular name	Gene name	AF ID	Score
11634725	VX.11e	PTN11	AF-Q06124	−9.6
60838	Irinotecan	PTN11	AF-Q06124	−9.7
457193	Dactinomycin	PTN11	AF-Q06124	−9.6

### 3.4. DEGs 1 and DEGs 2 affected the development of PTC in different ways

**Figure 4.**
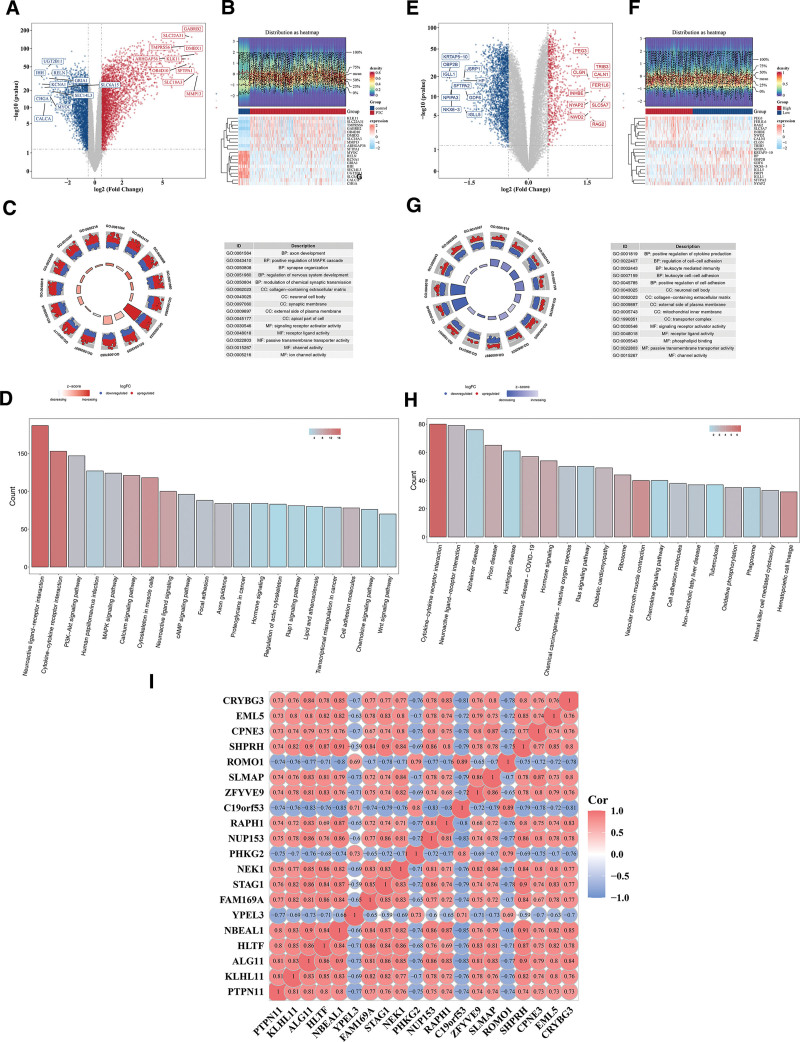
Confirmation of DEG1 and DEG2 and analysis with GO and KEGG. (A) Volcanic map of DEGs1. Blue: down. Red: up. (B) Heat map of DEGs1. Red: high expression; blue: low expression. (C) GO enrichment analysis of DEGs1. Red represented upregulated genes, blue represented downregulated genes. (D) KEGG enrichment analysis of DEGs1. Red to blue referred to the change in the value of −log_10_ (*P*-value). (E) Volcanic map of DEGs2. Blue: down. Red: up. (F) Heat map of DEGs 2. Red: high expression; blue: low expression. (G) GO enrichment analysis of DEGs2. Red represented upregulated genes, blue represented downregulated genes. (H) KEGG enrichment analysis of DEGs2. Red to blue referred to the change in the value of −log_10_ (*P*-value). (I) Heat map of the correlation for DEGs 1. Red: positive correlation. Blue: negative correlation. DEG = differentially expressed gene, GO = gene ontology, KEGG = Kyoto Encyclopedia of Genes and Genomes.

There were 5914 DEGs 1 between the PTC and control samples, including 3252 upregulated genes and 2662 downregulated genes (Fig. 4A, B). Subsequently, GO enrichment analysis was carried out for DEGs 1. A total of 2756 results were obtained, and 15 results were shown, including axon development, synapse organization, and receptor ligand activity (*P* < .05; Fig. 4C, Table S2, Supplemental Digital Content, https://links.lww.com/MD/Q823). Moreover, the pathways for KEGG enrichment were neuroactive ligand–receptor interaction, cytokine–cytokine receptor interaction, PI3K-Akt signaling pathway, etc (*P* < .05; Fig. 4D, Table S3, Supplemental Digital Content, https://links.lww.com/MD/Q823). Moreover, There were 3107 DEGs 2 between *PTPN11* HEG and LEG, including 727 upregulated genes and 2380 downregulated genes (Fig. 4E, F). Subsequently, GO enrichment analysis of DEGs 2. A total of 1216 results were obtained, and 15 results were shown. Among them, the cell components included neuronal cell body and collagen-containing extracellular matrix, and molecular functions included signaling receptor activator activity and phospholipid binding (*P* < .05; Fig. 4G, Table S4, Supplemental Digital Content, https://links.lww.com/MD/Q823). Moreover, the pathways for KEGG enrichment were neuroactive ligand–receptor interaction, cytokine–cytokine receptor interaction, Alzheimer disease, etc (*P* < .05; Fig. 4H, Table S5, Supplemental Digital Content, https://links.lww.com/MD/Q823). *PTPN11* was most pronounced negative correlation with YPEL3 in DEGs1 (cor = −0.75, *P* < .05), and most pronounced positive correlation with KLHL11 and ALG11 (cor = 0.81, *P* < .05; Fig. 4I).

### 3.5. A total of 8 genes that impact PTC prognosis were discovered

**Figure 5.**
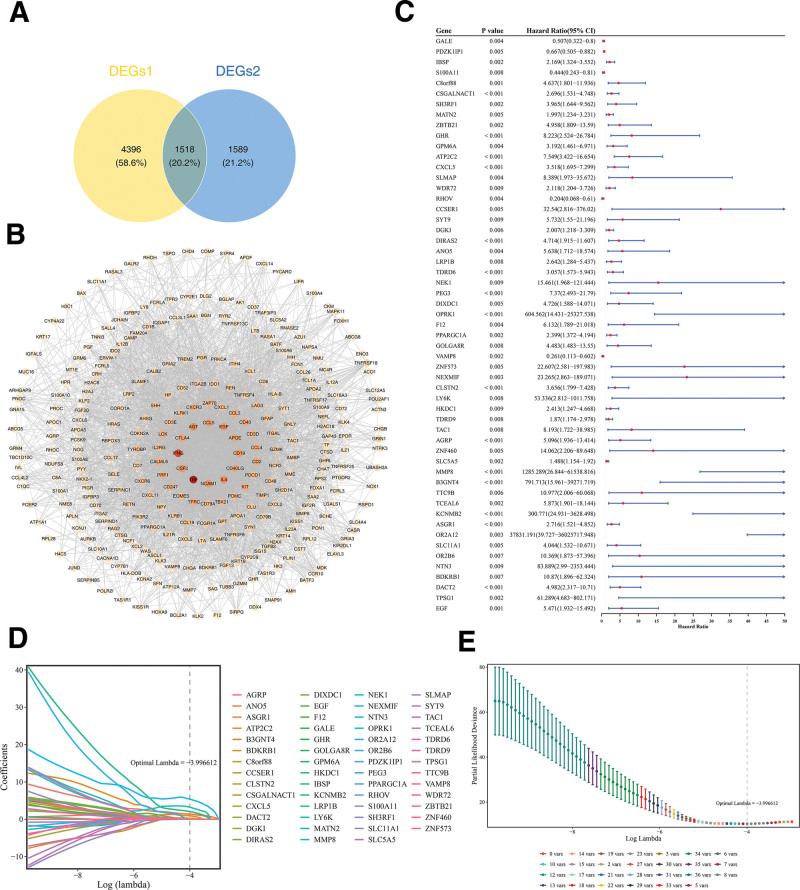
Determination of prognostic genes. (A) Venn diagram of candidate genes. About 1518 genes were confirmed as candidate genes. (B) PPI network diagram of candidate genes. Nodes represented genes, edges represented interactions between genes, and darker colors represented more interactions between this gene and other genes. (C) Univariate Cox analysis forest plot. From left to right are prognostic related genes, corresponding *P*-values, risk ratios, and their confidence intervals. The smaller the range of confidence intervals, the higher their credibility. (D) LASSO coefficient spectrum. Each line represented a gene. (E) Cross validation of adjustment parameters in LASSO analysis. Each line represented a gene. LASSO = least absolute shrinkage and selection operator, PPI = protein–protein interaction.

Furthermore, 5914 DEGs 1 and 3107 DEGs 2 were intersected, and 1518 intersection genes were obtained as candidate genes (Fig. 5A). In the PPI network, only the top 300 genes in degree ranking were shown. Among them, TNF had the most interactions with other genes (Fig. 5B).

Following univariate Cox regression analysis with criteria set as hazard ratio ≠ 1 and *P* < .01, accompanied by verification via the proportional hazards assumption test, where *P* > .05, a total of 55 genes were retained for subsequent analysis (Figs. 5C and S1, Supplemental Digital Content, https://links.lww.com/MD/Q822). Subsequently, when optimal lambda was equal to −3.996612, LASSO analysis screened 8 prognostic genes (*ATP2C2*, *OPRK1*, *CLSTN2*, *AGRP*, *MMP8*, *B3GNT4*, *KCNMB2*, and *DACT2*; Fig. 5D, E). So, they could be utilized to establish the final prognostic risk model.

### 3.6. A reliable prognostic risk model was established

**Figure 6.**
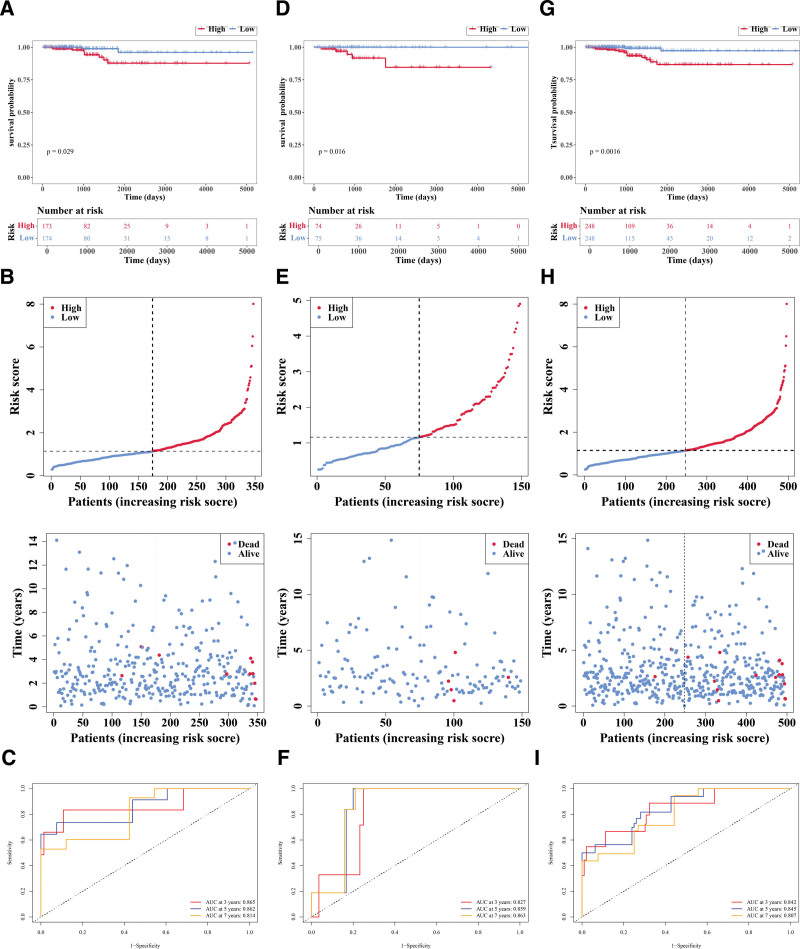
Construction and validation of the prognostic risk model. (A) Kaplan-Meier (K-M) survival curves of the high-risk group (HRG) and low-risk group (LRG) in the training set. Red: high-risk group; blue: low-risk group. The upper ordinate represents the survival proportion, and the lower ordinate represents the grouping. The abscissa denotes survival time, and the numbers in the grouping represent the number of surviving patients. *P* value indicates statistical significance. (B) Risk score distribution plot (upper) and survival status distribution plot (lower) of patients in the training set. In the risk score plot, red represents high risk and blue represents low risk; the ordinate is the risk score, and the abscissa is the patient. In the survival status plot, red represents dead and blue represents alive, with the dashed line indicating the median risk score. (C) Receiver operating characteristic (ROC) curves of HRG and LRG for 3 years, 5 years, and 7 years survival periods in the training set. The larger the area under the curve, the higher the AUC value, indicating a higher accuracy of the prognostic risk model in predicting patients’ survival status. The colors of the curves correspond to different years. The ordinate is sensitivity, and the abscissa is 1-specificity. (D) K–M survival curves of HRG and LRG in validation set 1. (E) Risk score distribution plot and survival status distribution plot of validation set 1. (F) ROC curves of HRG and LRG for 3 years, 5 years, and 7 years survival periods in validation set 1. (G) K-M survival curves of HRG and LRG in validation set 2. (H) Risk score distribution plot and survival status distribution plot of validation set 2. (I) ROC curves of HRG and LRG for 3 years, 5 years, and 7 years survival periods in validation set 2. AUC = area under curve, HRG = high-risk group, K–M = Kaplan–Meier, LRG = low-risk group, ROC = receiver operating characteristic.

Within the training set, patients were divided into HRG (n = 173) and LRG (n = 174) based on the median cutoff value (1.140948). Then the research found that there were substantial disparities in survival rate between HRG and LRG (*P* = .029; Fig. 6A); as risk scores increased, survival time decreased and more deaths occurred (Fig. 6B). The area under curve (AUC) value (3−(0.865), 5−(0.862), and 7−(0.814) years) indicated that this prognostic risk model functioned effectively in predicting the survival status (Fig. 6C). Then, in the validation set 1, patients were segmented into HRG (n = 74) and LRG (n = 75) in accordance with the median cutoff value (1.157863). Notably, significant differences in survival rates were observed between HRG and LRG (*P* = .016; Fig. 6D); as risk scores increased, survival time decreased and more deaths occurred (Fig. 6E). The AUC value (3−(0.827), 5−(0.859), and 7−(0.863) years) also indicated it performed well in predicting the survival status (Fig. 6F). Moreover, in the validation set 2, patients were segmented into HRG (n = 248) and LRG (n = 248) in accordance with the median cutoff value (1.145071). Similarly, marked differences in survival rates were observed between HRG and LRG (*P* = .0016; Fig. 6G); as risk scores increased, survival time decreased and more deaths occurred (Fig. 6H). The AUC value (3−(0.842), 5−(0.845), and 7−(0.807) years) also indicated it performed well in predicting the survival status (Fig. 6I). The above results indicate that the prognostic model based on 8 genes exhibits stable predictive performance across different cohorts and can serve as a potential independent assessment tool for overall survival in patients with PTC.

### 3.7. There were 8 cell types that were annotated altogether inGSE191288

**Figure 7.**
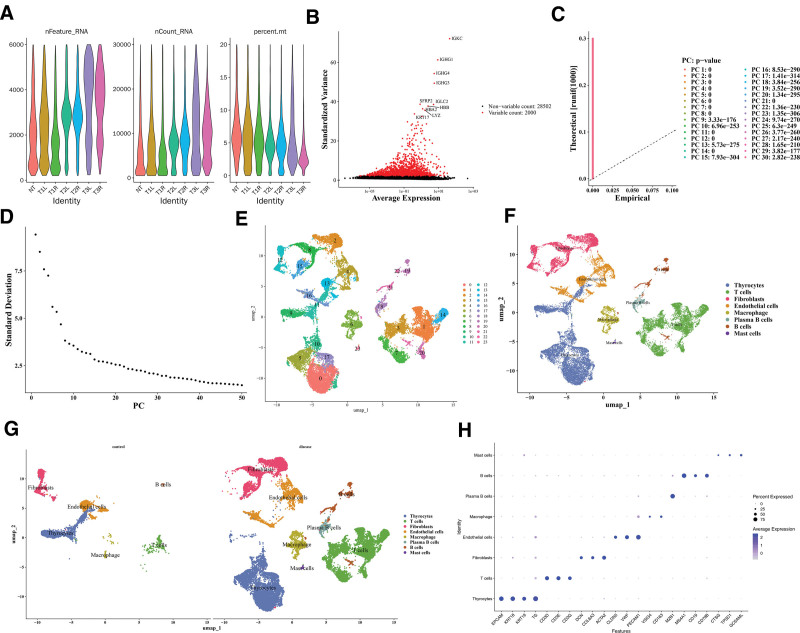
Cell annotation of GSE191288. (A) Single cell data quality control. The *x*-axis represented different cells. (B) Screening of highly variable genes. The red color in the legend represented highly variable genes and displayed the names of the top 10 genes. (C, D) Principal component inflection point diagram. (C) The more the curve deviated from the dashed line, the greater the difference between the distribution of principal components and the uniform distribution. Colors represented different principal components. (D) The horizontal axis represented the number of main components, and the vertical axis represented the standard deviation. (E) Cell UMAP clustering diagram. Different colors represented different clusters of cells. (F) Cell annotation results. Each color represented a type of cell. (G) Annotation results of cells in different groups. Each color represented a type of cell. (H) Cell cluster marker gene expression map. The dot color from blue to transparent represented the gene expression level from strong to weak. Size represented the proportion of gene expression levels.

Quality control was carried out for GSE191288, and after the removal of some cells, the nFeature_RNA, nCount_RNA, and percent_mt distribution maps after quality control were displayed (Fig. 7A). Thereafter, 2000 HVGs were identified and the 10 most important genes were labeled, such as *IGKC*, *IGHG1*, and *IGHG4* (Fig. 7B). The top 30 principal components with statistical significance were determined according to the inflection point of the Elbow plot for subsequent single-cell analysis (*P* < .05; Figs. 7C, D). As shown, cells were divided into 24 clusters (Fig. 7E); all clusters were annotated as 8 cell types, such as Thyrocytes, T cells, and Fibroblasts (Fig. 7F). PTC samples exhibited elevated plasma B cells and mast cells compared to controls, indicating intergroup differences in cell composition (Fig. 7G). The plot of marker gene expression in cells was shown (Fig. 7H).

### 3.8. Cells influenced the progression of tumor through interactions among cells

**Figure 8.**
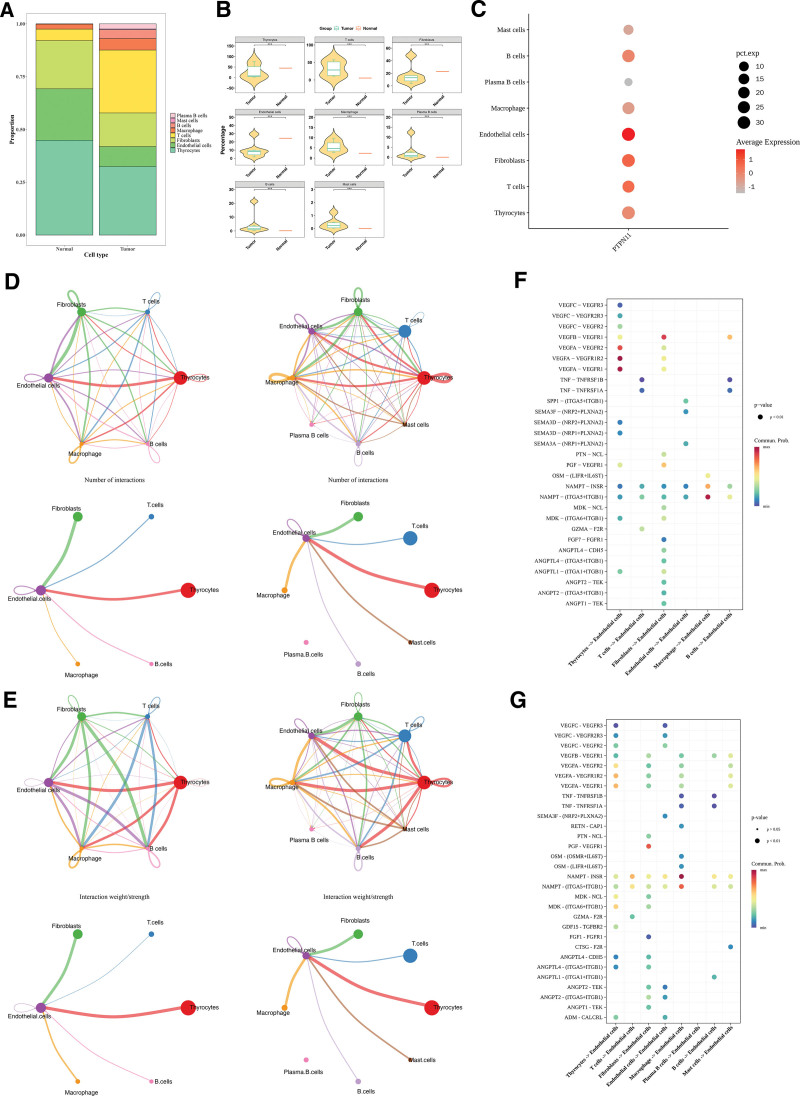
Analysis of intercellular interactions. (A) Proportion of various types of cell clusters. Different colors were different clusters of cells. (B) Significant differences in cell types between PTC sample tissue and normal tissue. ***P < .001. (C) Bubble plots of gene PTPN11 in different cell clusters. The larger and redder the circle, the higher the gene expression in the cell. (D) Network diagram of the number of connections between different cell clusters. The larger the interaction, the thicker the line. (E) Network diagram of connection weights between different cell clusters. The larger the interaction, the thicker the line. (F, G) Receptor ligand interactions between different cell types. The size of the dot represented the *P*-value, and the color of the dot represented the probability of communication. PTC = papillary thyroid carcinoma.

The distribution results of these 8 types of cells in the PTC and control samples is shown in Figure 8A. Box plots showed notable disparities in the proportions of all cells (*P* < .05; Fig. 8B). Additionally, endothelial cells were selected as key cells on the basis of the expression of *PTPN11* in these 8 cell types (Fig. 8C).

The cell communication network diagram showed that after the occurrence of PTC, the number of endothelial cells decreased, and the frequency and intensity of interactions between endothelial cells and mast cells were affected. The number of T cells increased, and the interaction intensity between endothelial cells and T cells was enhanced (Fig. 8D, E). Receptor–ligand analysis showed *VEGFA*-*VEGFR1R2* had the highest interaction frequency between endothelial and thyrocytes in controls. Among the tumor group, the communication frequency of *NAMPT*-*INSR* was the highest during the interaction between endothelial cells and macrophages (Fig. 8F, G). These results provided references for the pathological mechanism of PTC.

## 4. Discussion

The incidence of PTC continues to rise.^[26,27]^ The research on the etiology and progression of PTC is of great significance. *PTPN11*, a multifunctional tyrosine phosphatase, has been implicated in the onset and advancement of various malignancies.^[28]^ The present investigation primarily assesses the prognostic significance of the *PTPN11* gene across different cancers, including PTC, utilizing publicly available datasets and existing literature. This encompasses analyses such as GSEA, immune infiltration studies, drug sensitivity assessments, molecular docking, prognostic model development, and single-cell transcriptomic evaluations. These findings help us to understand the impact of *PTPN11* on the occurrence and development of PTC and provide new directions for the treatment and disease prognosis assessment of PTC.

In this study, we established a prognostic model by identifying 8 genes associated with overall survival in PTC patients for the first time. Higher risk scores correlate with poorer prognoses. These 8 genes were: *ATP2C2*, *OPRK1*, *CLSTN2*, *AGRP*, *MMP8*, *B3GNT4*, *KCNMB2*, and *DACT2. ATP2C2* is a secretory pathway calcium ion-transporting ATPase 2, belonging to the P-type ATPase family, encoding a calcium ion transport protein involved in the regulation of intracellular calcium homeostasis.^[29]^ A study suggests that *ATP2C2* may impede the migration and metastasis of cancer cells by counteracting the epithelial-mesenchymal transition.^[30]^ Moreover, patients exhibiting elevated *ATP2C2* expression demonstrate an increased mortality risk, positioning the prognostic gene model incorporating *ATP2C2* as an independent prognostic factor, thus providing improved survival predictions for patients with THCA,^[31]^ which is consistent with our study. *DACT2*, a member of the DACT (dapper) family, acts as a secreted glycoprotein and tumor-suppressor gene that modulates the Wnt/β-catenin pathway.^[32]^ The activation of the Wnt/β-catenin signaling pathway is often associated with carcinogenesis and influences the progression of hepatocellular carcinoma, colorectal cancer, and other types of cancer.^[33]^ In THCA, the *DACT2* gene is often methylated, affecting cancer occurrence and development through epigenetics.^[34]^ Furthermore, in PTC, *DACT2* is downregulated by *MLL3*, leading to *YAP*-*VEGF*-mediated tumor angiogenesis.^[35]^ *OPRK1*, *CLSTN2*, *AGRP*, *MMP8*, *B3GNT4*, and *KCNMB2* have been reported to be associated with various malignancies, but their relationship with PTC has rarely been reported.

By enrichment analysis, *PTPN11* was found to be significantly enriched in pathways such as ribosome, OXPHOS, and Parkinson’s disease. Research has found that *TERT* accelerates the dedifferentiation and progression of *BRAF* mutant-induced THCA by regulating ribosome biogenesis^[36]^ (studies indicate that *focal adhesion kinase* can drive the growth, invasion, and metastasis of THCA, promoting ribosome biogenesis, thereby driving the growth and survival of advanced THCA cells.^[37]^ Prognostic studies in PTC have shown higher levels of OXPHOS in the tumor mutational burden group.^[38]^ In most PTCs, OXPHOS complex I is significantly decreased (cells 2018, 7, 40^[39]^). Future interventions targeting these pathways may help address the progression of PTC.

This research identified notable variations in 21 types of immune cells between high and low expression cohorts of *PTPN11*, including activated B cells and activated CD8 T cells, with the majority demonstrating positive correlations. Notably, a significant negative correlation was observed between *PTPN11* and *CD56* positive NK cells. Studies indicate that regulatory T cells, neutrophils, and dendritic cells play tumor-promoting roles in the PTC tumor microenvironment, while CD8+ T cells, B cells, and NK cells act as protective factors.^[40,41]^ The onset and progression of PTC are strongly associated with inflammation and immune cell infiltration.^[42]^ Studies have demonstrated that M2 macrophages, Tregs, monocytes, neutrophils, DCs, MCs, and M0 macrophages exert pro-tumor effects, whereas M1 macrophages, CD8+ T cells, B cells, NK cells, and T follicular helper cells play antitumor roles.^[43]^ Consistent with previous studies, the outcomes of this study also suggested that prognostic genes might influence the immune cell infiltration of PTC, and this might provide a reference for the clinical management of PTC.

Molecular docking and drug sensitivity analysis show that *PTPN11* (SHP2) has good binding ability with drugs VX-11e, irinotecan, and dactinomycin. The MAPK signaling pathway is pivotal in THCA, with ERK kinases representing crucial downstream targets. VX-11e is a small molecule ERK inhibitor. Previous studies have found that in mouse models, the use of VX-11e alone can inhibit tumor tissue growth, when combined with the PI3K inhibitor BKM120, it can significantly inhibit tumor growth.^[44]^ Irinotecan is a chemotherapy drug commonly used to treat colorectal cancer and colon cancer, blocking cancer cell growth and proliferation by inhibiting the activity of DNA topoisomerase I.^[44]^ Dactinomycin is an anticancer drug commonly used to treat various malignant tumors.^[45]^ The results suggest that these drugs may have the opportunity to be applied to PTC patients.

Eight cell types were annotated in single-cell analysis of this study, such as thyroid cells, T cells, B cells, and fibroblasts, and selected endothelial cells as key cells based on the expression level of the *PTPN11* gene in cells. Cell communication analysis found that the number of endothelial cells decreased in PTC, with more interactions and stronger interaction intensity between endothelial cells and mast cells. The number of T cells increased, and the interaction intensity between endothelial cells and T cells was enhanced. Studies have revealed that endothelial cells are associated with the progression of PTC.^[46]^ Additionally, studies have revealed high infiltration of fibroblasts in PTC and their interactions with various cell types, revealing differentially expressed fibroblast-related genes in THCA tissues.^[47]^ CD8+ T cells have strong cytotoxicity and are the main executors of antitumor immune function. Studies have observed a large infiltration of CD8+ T cells in PTC,^[48]^ which aligns with the findings of the present study.

This study was conducted based on public databases, including TCGA and gene expression omnibus, to preliminarily explore the prognostic potential of PTPN11 in PTC and to construct a prognostic model based on potential prognostic genes. However, due to limitations in data sources and study design, there are notable constraints. The conclusions rely solely on public data, and the expression and regulatory mechanisms of PTPN11 and the prognostic genes – such as the role of PTPN11 in ribosomal and OXPHOS pathways – have not been validated in clinical samples. The model was developed using only the TCGA-THCA cohort and lacks validation of its generalizability in combination with clinicopathological features. Furthermore, the associations between PTPN11 and immune cell infiltration, drug interactions, as well as its interplay with prognostic genes, require further substantiation through in vitro and in vivo experiments. In future work, we plan to collect 150 PTC clinical tissue samples from 3 tertiary hospitals and validate gene expression and mechanisms using IHC and qRT-PCR. A multicenter cohort of over 500 cases will be established in collaboration with 5 to 8 medical centers to optimize a combined “gene-clinical” predictive model. Additionally, PTPN11-overexpressing and knockdown PTC cell lines, as well as xenograft tumor models in nude mice, will be constructed. These will be combined with drug IC_50_ assays to strengthen the study conclusions and provide a foundation for clinical translation.

## 5. Conclusion

Based on public datasets, this study explored the prognostic potential of *PTPN11* in PTC from various aspects and screened 8 related prognostic genes, successfully constructing a prognostic risk model. However, the current study still has some limitations. Due to the complexity and diversity of biological systems, it may not fully reflect the actual biological processes, and future studies are required to verify their clinical significance.

## Acknowledgments

We would like to express our sincere gratitude to all individuals and organizations who supported and assisted us throughout this research. Special thanks to the self-funded scientific research project of the Health Commission of Guangxi Zhuang Autonomous Region. In conclusion, we extend our thanks to everyone who has supported and assisted us along the way. Without your support, this research would not have been possible.

## Author contributions

**Conceptualization**: Huiling Wang, Huaye Lao, Minghui Qin, Hui Zhang, Guixuan Nong, Yinling Wang.

**Data curation**: Huiling Wang, Mian Lv, Yonghong Huang, Xiaoming Pan.

**Project administration**: Hui Zhang, Guixuan Nong, Yinling Wang.

**Supervision**: Hui Zhang, Guixuan Nong, Yinling Wang.

**Visualization**: Huiling Wang, Mian Lv, Yonghong Huang, Xiaoming Pan, Chunyu Chen, Wuyu Tan, Huaye Lao, Minghui Qin.

**Validation**: Huiling Wang, Mian Lv, Yonghong Huang, Xiaoming Pan, Changqiang Xu.

**Writing – original draft**: Huiling Wang.

**Writing – review & editing**: Huiling Wang, Mian Lv, Yonghong Huang, Xiaoming Pan, Changqiang Xu, Chunyu Chen, Wuyu Tan, Huaye Lao, Minghui Qin, Hui Zhang, Guixuan Nong, Yinling Wang.

## Supplementary Material

**Figure:**